# Global burden of nonalcoholic steatohepatitis-related liver cancer, 1990–2019: a systematic analysis for the GBD 2019

**DOI:** 10.1186/s13098-022-00885-y

**Published:** 2022-08-09

**Authors:** Juan Pang, Ke Chen, Shen Chen, Xu Chen

**Affiliations:** 1 Department of Nutrition, School of Public Health, Sun Yat-Sen University (Northern Campus), No.74, 2nd Zhongshan Road, Guangzhou, People’s Republic of China 510080; 2 Department of Toxicology, School of Public Health, Sun Yat-Sen University (Northern Campus), No.74, 2nd Zhongshan Road, Guangzhou, People’s Republic of China 510080; 3 Department of Molecular, Cellular, and Developmental Biology, University of Colorado, Boulder, USA

**Keywords:** Global Burden of Disease, Nonalcoholic fatty liver disease, Liver cancer, Nonalcoholic steatohepatitis

## Abstract

With the pandemic of metabolic diseases, nonalcoholic fatty liver disease (NAFLD) prevalence has dramatically elevated. NAFLD encompasses a spectrum of diseases including simple steatosis and nonalcoholic steatohepatitis (NASH), which can further progress to cirrhosis or liver cancer (LC). However, data are lacking on the burden and trend of NASH-related LC. Here, we analyzed the trends and changes of NASH-related LC burden using Global Burden of Disease (GBD) data (1990–2019). In 2019, the global incidence, prevalence, disability-adjusted life years (DALYs) and deaths of NASH related LC were 36.3 thousand (95% UI 29.5–44.9), 46.8 thousand (38.2–57.6), 796 thousand (657–976) and 34.7 thousand (28.4–43.2), respectively. The absolute numbers and rates of NASH-related LC incidence, mortality, and DALY significantly elevated from 1990 to 2019. With the age increased, the incidences, DALYs and deaths of NASH-related LC significantly elevated. The incidence and mortality rate of NASH-related LC significantly increased from 2010 to 2019 in individuals aged from 20 to 54 and older than 55 years old. We also found that a large disparity of NASH-related LC burden in different socio-demographic index (SDI) locations. The crude number and the age-standardized rate of incidences, DALYs and deaths was highest in the middle SDI locations and high SDI locations showed the largest increase of NASH-related LC burden from 1990 to 2019. Moreover, the proportion of LC incidences, deaths and DALYs attributed to NASH were 4.74%, 5.30% and 4.25%, respectively in 1990 which were increased by 43.5%, 35.3% and 49.4%, respectively in 2019. Conclusion: The global burden of NASH-related LC and the proportion LC burden attributed to NASH are significantly increasing.

## Introduction

Nonalcoholic fatty liver disease (NAFLD) encompasses a spectrum of diseases including simple steatosis which has a benign clinical course, and nonalcoholic steatohepatitis (NASH), which can further progress to advanced liver diseases including cirrhosis and hepatocellular carcinoma (HCC) [1, 2]. NASH presents lipotoxicity, chronic inflammation, fibrosis and even cirrhosis in the liver, thus leading to a pro-carcinogenic state [3]. It is estimated that 25% of the general US population was affected by the NAFLD, among which 20 ~ 25% will progress to NASH and 4 ~ 11.3% NASH patients ended up in liver cancer (LC) [4–6]. Paralleling the increase of diabetes and metabolic syndrome, NASH prevalence has increased dramatically worldwide during the recent years and is projected to elevate by up to 56% between 2016 and 2030 [7].

Liver cancer is one of the most dominant lethal malignancies globally, which was estimated to cause 12.5 million disability-adjusted life-years and lead to approximately 485 thousand deaths in 2019 [8]. The present main causes of LC are still the hepatitis B virus (HBV) and hepatitis C virus (HCV) infection. However, with the availability of vaccination and antiviral drugs for HBV and HCV, the burden of chronic viral liver disease is decreasing in recent years [9, 10]. On the other side, there is no approved therapy for NASH to date [11]. Given that a larger proportion of LC that arise in NASH tends to occur in the absence of liver cirrhosis and is associated with worse survival outcomes than HBV or HCV-related LC, the disease burden of NASH-related LC is largely underestimated and needs to be updated [12–14].

The Global Burden of Disease Study (GBD) provides a unique framework to examine disease burden, using vital registry information, health administrative reports, disease surveillance, and other sources [15]. The GBD data makes policymakers fully understand what the true health challenges are and how these challenges change over time, thus providing fundamental guidance to disease prevention and control. Here, in this study, GBD 2019 was used to analyze the incidence, prevalence, death, and disability-adjusted life years (DALYs) of NASH-related LC worldwide from 1990 to 2019, stratified by sex, age, socioeconomic development status.

## Methods

The GBD 2019 data was used in this study, which is collected from the Global Health Data Exchange query tool (https://ghdx.healthdata.org). The GBD 2019 was coordinated by the Institute for Health Metrics and Evaluation (IHME). Total 204 countries and territories data of 256 causes of death, 369 diseases and injuries were available [15]. The countries and territories were divided into 5 categories based on the socio-demographic index (SDI). The methods of calculating SDI are described in detail in a previous study [16]. The value of SDI ranges from 0 to 1. A specific country or territory with an SDI higher than 80th percent, 60th–79th, 40th–59th, 20th–39th and lower than 20th of the ranked SDI values was categorized as high, high-middle, middle, low-middle, and low SDI locations, respectively. The methods for estimations of LC burden have been reported in detail [8]. Briefly, the cancer burden information in GBD was sought from multiple individual national cancer registries or aggregate database of cancer registries, such as NORDCAN, Cancer Incidence in Five Continents (CI5), SEER or EUEGR. ICD codes used were C22.0–9. Because the ICD-10 coding is unsuitable for liver cancer etiological estimates, the GBD team split the five causes (HBC, HCV, ALD, NASH, and other causes) using the etiological proportions estimated by DisMod-MR 2.1, a Bayesian meta-regression model. Here, we investigated the disease burden of liver cancer caused by NASH in detail. We used four standard epidemiological measures to assess the global burden of NASH-related LC including deaths, prevalence, incidence, and disability-adjusted life-years (DALYs). DALYs is an index that represents health loss from both fatal and non-fatal outcomes, equal to years lived with disability (YLDs) plus years of life lost (YLLs) [17]. Age-standardized rates for incidence, mortality, prevalence, and DALYs were calculated by the age structure from 2019. The uncertainty interval (UI) analysis was used in the GBD to address the possible heterogeneity from both sampling error and non-sampling variance. The 95% UIs were calculated by taking 1000 samples from the posterior distribution of the respective step in the modelling process and reported as the 2.5th and 97.5th values for each estimate [18]. The University of Washington Institutional Review Board Committee approved the Global Burden of Diseases, Injuries, and Risk Factors Study (STUDY00009060).

## Results

### Global burden of NASH-related LC

In 2019, the global incidence, prevalence, DALYs and deaths of NASH related LC were 36.3 thousand (95% UI 29.5–44.9), 46.8 thousand (38.2–57.6), 796 thousand (657–976) and 34.7 thousand (28.4–43.2), respectively. There was an age-standardized incidence rate of 0.448 (0.365–0.554) and an age-standardized prevalence rate of 0.573 (0.467–0.705) per 100,000 population respectively for the NASH-related LC. For the age-standardized DALY rates, the global rate was 9.64 (7.98–11.8) per 100,000 person-years for NASH-related LC in 2019. Moreover, there was an age-standardized death rate of 0.431 (0.353–0.534) per 100,000 population for NASH-related LC in 2019. As shown in Table 1, the incidence, prevalence, DALYs and deaths were higher in male than female subjects globally.

**Table 1:** Number, rate, and age-standardised rate for NASH-related HCC incidences, prevalence, DALYs and deaths in 2019

	Male	Female	Total
*Incidence*			
Number, in thousands	18.6 (14.9, 23.5)	17.7 (14.0, 22.0)	36.3 (29.5, 44.9)
Rate, per 100,000	0.479 (0.385, 0.607)	0.460 (0.364, 0.570)	0.470 (0.381, 0.580)
Age-standardised rate, per 100,000	0.493 (0.395, 0.622)	0.407 (0.322, 0.503)	0.448 (0.365, 0.554)
*Prevalence*			
Number, in thousands	24.6 (19.9, 30.9)	22.2 (17.7, 27.3)	46.8 (38.2, 57.6)
Rate, per 100,000	0.635 (0.512, 0.796)	0.575 (0.460, 0.709)	0.605 (0.494, 0.744)
Age-standardised rate, per 100,000	0.641 (0.518, 0.805)	0.512 (0.410, 0.629)	0.573 (0.467, 0.705)
*DALYs*			
Number, in thousands	417 (336, 520)	378 (309, 471)	796 (657, 976)
Rate, per 100,000	10.8 (8.65, 13.4)	9.81 (8.00, 12.2)	10.3 (8.49, 12.6)
Age-standardised rate, per 100,000	10.6 (8.50, 13.2)	8.76 (7.15, 10.9)	9.64 (7.98, 11.8)
*Deaths*			
Number, in thousands	17.4 (14.0, 22.0)	17.4 (13.8, 21.8)	34.7 (28.4, 43.2)
Rate, per 100,000	0.448 (0.360, 0.567)	0.450 (0.358,0.565)	0.449 (0.367, 0.558)
Age-standardised rate, per 100,000	0.470 (0.380, 0.593)	0.397 (0.317, 0.499)	0.431 (0.353, 0.534)

Data are presented as value (95% uncertainty interval)

### Temporal changes in burden of NASH-related LC

The temporal changes of global NASH-related LC burden were shown in Table 2. The incident cases of NASH-related LC increased by 42.0% (23.6%, 62.6%) from 1990 to 2019. And the age-standardized incidence rate had a larger increase from 2010 to 2019 (8.16%, [0.0114%, 17.0%]) than from 1990 to 2019 (1.19% [− 11.4%, 15.5%]). The number of deaths from NASH-related LC increased by 95.1% (70.2%, 123%) from 1990 to 2019. The crude mortality rate of NASH-related LC increased by 34.9% (17.7%, 54.1%) from 1990 to 2019, as did that by 24.2% (15.3%, 33.5%) from 2010 to 2019. Meanwhile, the DALYs of NASH-related LC increased by 66.2% (95% UI 43.9% to 91.1%), from 478.9 thousand (392.6 to 577.8) in 1990 to 795.8 thousand (657.3 to 975.8) in 2019. Despite the DALYs of NASH-related LC having been increasing steadily since 1990, we found a substantial reduction (− 15.1% [− 26.1 to − 2.62]) in the age-standardized DALYs from 1990 to 2019, suggesting that ageing is the major driver of the increase.

**Table 2:** Percentage changes for NASH-related HCC incidence, DALYs and deaths from 1990 or 2010 to 2019

	Male	Female	Total
*Incidence*			
Number (percentage change, 1990–2019)	121% (88.5%, 161%)	90.9% (57.0%, 127%)	105% (78.8%, 135%)
Number (percentage change, 2010–2019)	44.1% (30.3%, 59.1%)	33.2% (20.7%, 46.1%)	38.6% (27.9%, 50.0%)
Rate (percentage change, 1990–2019)	53.6% (30.8%, 81.3%)	31.5% (8.12%, 56.1%)	42.0% (23.6%, 62.6%)
Rate (percentage change, 2010–2019)	30.4% (17.9%, 43.9%)	20.1% (8.80%, 31.7%)	25.2% (15.5%, 35.4%)
Age-standardised rate (percentage change, 1990–2019)	8.94% (− 6.59%, 27.4%)	− 5.83% (− 22.3%, 11.9%)	1.19% (− 11.4%, 15.5%)
Age-standardised rate (percentage change, 2010–2019)	12.2% (1.92%, 23.5%)	4.37% (− 4.94%, 14.6%)	8.16% (0.0114%, 17.0%)
*DALYs*			
Number (percentage change, 1990–2019)	78.8% (50.3%, 109%)	54.1% (25.6%, 84.7%)	66.2% (43.9%, 91.1%)
Number (percentage change, 2010–2019)	38.4% (24.7%, 52.5%)	28.6% (15.5%, 42.3%)	33.6% (23.0%, 44.3%)
Rate (percentage change, 1990–2019)	24.1% (4.29%, 45.6%)	6.16% (− 13.5%, 27.2%)	14.9% (− 0.498%, 32.1%)
Rate (percentage change, 2010–2019)	25.3% (12.8%, 38.0%)	15.9% (4.12%, 28.2%)	20.6% (11.1%, 30.3%)
Age-standardised rate (percentage change, 1990–2019)	− 8.72% (− 22.8%, 6.55%,)	− 21.2% (− 35.6%, − 5.31%)	− 15.1% (− 26.1%, − 2.62%)
Age-standardised rate (percentage change, 2010–2019)	10.1% (− 0.478%, 21.1%)	2.65% (− 7.80%, 13.3%)	6.37% (− 1.82%, 14.6%)
*Deaths*			
Number (percentage change, 1990–2019)	109% (77.6%, 142%)	82.9% (51.0%, 116%)	95.1% (70.2%, 123%)
Number (percentage change, 2010–2019)	42.9% (30.2%, 55.9%)	32.5% (20.3%, 44.8%)	37.5% (27.6%, 47.9%)
Rate (percentage change, 1990–2019)	45.1% (23.3%, 68.0%)	26.0% (4.02%, 49.1%)	34.9% (17.7%, 54.1%)
Rate (percentage change, 2010–2019)	29.3% (17.8%, 41.1%)	19.4% (8.45%, 30.5%)	24.2% (15.3%, 33.5%)
Age-standardised rate (percentage change, 1990–2019)	1.32% (− 12.9%, 16.3%)	− 11.1% (− 26.2%, 4.70%)	− 5.27% (− 16.7%, 7.21%)
Age-standardised rate (percentage change, 2010–2019)	10.5% (1.15%, 20.0%)	3.12% (− 6.50%, 12.5%)	6.64% (− 0.811%, 14.3%)

Data are presented as value (95% uncertainty interval)

### Burden of NASH-related LC in different age groups

We divided the population into three groups which are younger than 20 years old, 20–54 years old and older than 55 years old and further analyze the burden in these three groups. The incidence of NASH-related LC was much higher in older age group. In 2019, the global incidence of NASH-related LC was 0.240 thousand (95% UI 0.175–0.325), 6.13 thousand (4.64–8.12) and 30.0 thousand (23.7–37.9) in the age group younger than 20 years old, 20–54 years old and older than 55 years old, respectively (Table 3). The incidence increased significantly in the age group of 20–54 years old from 4.38 thousand (3.44 to 5.55) in 1990 to 6.13 thousand (4.64 to 8.12) in 2019. And we also found a substantial increase (14.3% [2.90%, 27.7%]) in the incidence rate of 20–54 years old group from 2010 to 2019 (Table 4).

**Table 3:** Number and rate for NASH-related HCC incidence, DALYs and deaths in different age groups in 2019

	Incidence		DALYs		Deaths	
	Number, in thousands	Rate, per 100,000	Number, in thousands	Rate, per 100,000	Number, in thousands	Rate, per 100,000
* < 20 years*						
Male	0.102 (0.0710, 0.145)	0.00769 (0.00534, 0.109)	4.40 (3.03, 6.29)	0.331 (0.228, 0.473)	0.0611 (0.0422, 0.0875)	0.00460 (0.00317, 0.00659)
Female	0.138 (0.101, 0.187)	0.0110 (0.00804, 0.0150)	5.89 (4.27, 8.07)	0.471 (0.342, 0.646)	0.0819 (0.0594, 0.112)	0.00655 (0.00476, 0.00898)
Total	0.240 (0.175, 0.325)	0.00931 (0.00680, 0.0126)	10.3 (7.53, 13.9)	0.399 (0.292, 0.539)	0.143 (0.105, 0.193)	0.00555 (0.00406, 0.00750)
*20–54 years*						
Male	3.43 (2.53, 4.56)	0.181 (0.134, 0.241)	120 (90.8, 162)	6.35 (4.80, 8.59)	2.76 (2.05, 3.79)	0.146 (0.109, 0.200)
Female	2.70 (2.04, 3.57)	0.145 (0.110, 0.192)	96.7 (73.0, 128)	5.19 (3.92, 6.88)	2.17 (1.64, 2.90)	0.117 (0.0880, 0.156)
Total	6.13 (4.64, 8.12)	0.163 (0.124, 0.216)	217 (167, 283)	5.77 (4.45, 7.54)	4.93 (3.77, 6.58)	0.131 (0.100, 0.175)
*55 + years*						
Male	15.1 (11.7, 19.5)	2.28 (1.76, 2.95)	293 (226, 385)	23.4 (18.1, 30.8)	14.6 (11.4, 18.9)	2.20 (1.72, 2.86)
Female	14.9 (11.6, 18.9)	2.00 (1.56, 2.54)	276 (217, 358)	18.5 (14.6, 24.0)	15.1 (11.9, 19.2)	2.03 (1.59, 2.58)
Total	30.0 (23.7, 37.9)	2.13 (1.69, 2.70)	569 (449, 731)	20.8 (16.4, 26.7)	29.7 (23.5, 37.7)	2.11 (1.67, 2.68)

Data are presented as value (95% uncertainty interval)

**Table 4:** Percentage changes for NASH-related HCC incidence, DALYs and deaths from 1990 or 2010 to 2019 in different age groups

	< 20 years	20–54 years	55 + years
*Incidence*			
Number (percentage change, 1990–2019)	9.03% (− 10.8%, 31.3%)	40.0% (17.4%, 66.1%)	129% (99.1%, 163%)
Number (percentage change, 2010–2019)	11.5% (− 0.986%, 26.5%)	25.7% (13.2%, 40.5%)	41.8% (31.5%, 53.3%)
Rate (percentage change, 1990–2019)	− 3.89% (− 21.4%, 15.8%)	− 10.3% (− 24.7%, 6.47%)	9.22% (− 4.94%, 25.5%)
Rate (percentage change, 2010–2019)	8.01% (− 4.12%, 22.5%)	14.3% (2.90%, 27.7%)	8.91% (0.971%, 17.7%)
*DALYs*			
Number (percentage change, 1990–2019)	2.87% (− 16.5%, 25.6%)	23.1% (4.05%, 46.0%)	94.2% (70.0%, 123%)
Number (percentage change, 2010–2019)	10.2% (− 2.08%, 26.5%)	21.8% (9.51%, 34.0%)	39.3% (29.4%, 49.8%)
Rate (percentage change, 1990–2019)	− 9.32% (− 26.5%, 10.7%)	− 21.1% (− 6.43%, − 33.3%)	− 11.2% (− 22.3%, 2.15%)
Rate (percentage change, 2010–2019)	6.76% (− 5.18%, 22.5%)	10.7% (− 0.475%, 21.8%)	5.86% (− 1.60%, 13.9%)
*Deaths*			
Number (percentage change, 1990–2019)	2.87% (− 16.6%, 25.7%)	26.2% (6.51%, 50.0%)	116% (88.6%, 147%)
Number (percentage change, 2010–2019)	10.3% (− 2.09%, 26.6%)	23.1% (10.6%, 35.6%)	40.5% (31.0%, 50.3%)
Rate (percentage change, 1990–2019)	− 9.33% (− 26.5%, 10.8%)	− 19.1% (− 31.7%, − 3.88%)	2.93% (− 9.98%, 17.9%)
Rate (percentage change, 2010–2019)	6.76% (− 5.20%, 22.3%)	11.8% (0.558%, 23.2%)	7.86% (0.632%, 15.4%)

Data are presented as value (95% uncertainty interval)

In 2019, there was 29.7 (95% UI 23.5–37.7) thousand people older than 55 years old died due to NASH-related LC. The mortality rate of NASH-related LC is highest in the population older than 55 years old. The NASH-related LC mortality rate increased by 7.86% (0.632%, 15.4%) in the age group of older than 55 years old from 1.96 (1.56 to 2.44) in 2010 to 2.11 (1.67 to 2.68) per 100, 000 population in 2019. The number of people aged 20–54 years old died due to NASH-related LC was 4.93 (3.77, 6.58) thousand. And the mortality rate of NASH-related LC increased by 11.8% (0.558%, 23.2%) from 2010 to 2019 in the age group of 20–54 years old. The people younger than 20 years old had quite lower NASH-related LC mortality [0.240 thousand (0.175, 0.325)] compared to other age groups.

As for DALYs, in 2019, there were 10.3 (7.53, 13.9), 217 (167, 283) and 569 (449, 731) thousand DALYs of NASH-related LC in the age group of younger than 20 years old, 20–54 years old and older than 55 years old, respectively. Moreover, DALYs of NASH-related LC increased by 23.1% (4.05%, 46.0%) in the age group of 20–54 years old and by 94.2% (70.0%, 123%) in the age group of older than 55 years old. However, it is worth noting that DALY rate of NASH-related LC decreased by 21.1% (6.43%, 33.3%) in the age group of 20–54 years old from 1990 to 2019.

### Burden of NASH-related LC in different socioeconomic development status

In 2019, stratified by SDI, the number and age-standardized incidence, death, and DALY rates for NASH-related LC were the highest for the middle SDI locations followed by the high SDI locations while the low SDI locations had the lowest rates (Table 5). The incident cases of NASH-related LC increased in all SDI locations from 1990 to 2019, with the largest increase for high SDI locations (234% [198–269]), followed by low SDI locations (124% [93.1, 160]), low to middle SDI locations (119% [87.7–153]), middle SDI locations (78.6% [48.0, 117]) and high to middle SDI locations (37.3% [16.9–60.6]) (Table 6). The deaths and DALYs number show the same trend among these five SDI locations. However, among different SDI locations, the age-standardized incidence, DALYs and mortality rates of NASH-related LC similarly decreased in middle and high to middle SDI locations but increased in high SDI locations from 1990 to 2019.

**Table 5:** Number and rate for NASH-related HCC incidence, prevalence, DALYs and deaths in different socioeconomic development status in 2019

	Low SDI	Low to middle SDI	Middle SDI	High to middle SDI	High SDI
*Incidence*					
Number, in thousands	1.75 (1.34, 2.27)	4.64 (3.75, 5.74)	13.9 (11.1, 17.5)	6.36 (5.19, 7.87)	9.65 (7.60, 12.3)
Rate, per 100,000	0.155 (0.119, 0.201)	0.263 (0.213, 0.325)	0.581 (0.462, 0.729)	0.445 (0.363, 0.550)	0.953 (0.750, 1.21)
Age-standardised rate, per 100,000	0.344 (0.263, 0.447)	0.348 (0.280, 0.429)	0.573 (0.458, 0.719)	0.316 (0.259, 0.388)	0.508 (0.406, 0.645)
*DALYs*					
Number, in thousands	49.7 (37.5, 63.9)	122 (98.3, 151)	333 (269, 418)	137 (113, 169)	154 nnn
Rate, per 100,000	4.41 (3.32, 5.66)	6.89 (5.57, 8.55)	13.9 (11.2, 17.4)	9.58 (7.88, 11.8)	15.2 (12.2, 19.3)
Age-standardised rate, per 100,000	8.50 (6.47, 11.1)	8.48 (6.85, 10.5)	13.0 (10.5, 16.3)	6.86 (5.68, 8.37)	8.70 (7.05, 10.8)
*Deaths*					
Number, in thousands	1.80 (1.37, 2.37)	4.84 (3.89, 6.06)	13.7 (10.9, 17.3)	6.15 (4.98, 7.56)	8.24 (6.48, 10.4)
Rate, per 100,000	0.160 (0.121, 0.210)	0.275 (0.220, 0.344)	0.571 (0.457, 0.722)	0.430 (0.348, 0.529)	0.813 (0.640, 1.02)
Age-standardised rate, per 100,000	0.375 (0.285, 0.487)	0.376 (0.302, 0.469)	0.578 (0.466, 0.728)	0.304 (0.248, 0.374)	0.419 (0.334, 0.526)

Data are presented as value (95% uncertainty interval)

**Table 6:** Percentage changes for NASH-related HCC incidence, DALYs and deaths from 1990 or 2010 to 2019 in different socioeconomic development status

	Low SDI	Low to middle SDI	Middle SDI	High to middle SDI	High SDI
*Incidence*					
Number (percentage change, 1990–2019)	124% (93.1%, 160%)	119% (87.7%, 153%)	78.6% (48.0%, 117%)	37.3% (16.9%, 60.6%)	234% (198%, 269%)
Number (percentage change, 2010–2019)	35.9% (24.9%, 48.4%)	45.3% (30.1%, 61.6%)	51.4% (33.6%, 69.8%)	31.7% (18.3%, 46.1%)	24.1% (13.2%, 35.4%)
Rate (percentage change, 1990–2019)	4.70% (− 9.64%, 21.9%)	40.3% (20.2%, 62.3%)	27.9% (6.05%, 55.4%)	10.4% (− 6.01%, 29.2%)	171% (142%, 199%)
Rate (percentage change, 2010–2019)	8.52% (− 0.306%, 18.5%)	29.0% (15.6%, 43.6%)	39.3% (22.9%, 56.2%)	24.9% (12.1%, 38.5%)	17.0% (6.71%, 27.7%)
Age-standardised rate (percentage change, 1990–2019)	0.116% (− 12.9%, 15.2%)	− 2.20% (− 16.7%, 14.0%)	− 23.3% (− 35.8%, − 7.30%)	− 27.1% (− 37.7%, − 15.2%)	82.9% (64.9%, 101%)
Age-standardised rate (percentage change, 2010–2019)	0.493% (− 7.01%, 9.44%)	10.0% (− 1.34%, 22.9%)	13.4% (0.472%, 26.8%)	5.98% (− 4.57%, 17.6%)	2.02% (− 7.33%, 11.6%)
*DALYs*					
Number (percentage change, 1990–2019)	130% (96.3%, 171%)	102% (73.2%, 134%)	56.8% (29.6%, 90.1%)	10.2% (− 6.08%, 26.0%)	155% (136%, 173%)
Number (percentage change, 2010–2019)	35.6% (23.0%, 49.3%)	40.9% (26.9%, 56.0%)	42.4% (25.3%, 61.1%)	24.3% (12.2%, 36.9%)	20.1% (12.7%, 26.7%)
Rate (percentage change, 1990–2019)	7.83% (− 8.17%, 26.9%)	29.5% (10.9%, 49.9%)	12.3% (− 7.16%, 36.1%)	− 11.4% (− 24.5%, 3.75%)	107% (91.1%, 122%)
Rate (percentage change, 2010–2019)	8.29% (− 1.81%, 19.2%)	25.2% (12.7%, 38.5%)	30.9% (15.2%, 48.2%)	17.8% (6.39%, 29.7%)	13.3% (6.26%, 19.5%)
Age-standardised rate (percentage change, 1990–2019)	3.20% (− 11.2%, 19.8%)	− 5.59% (− 19.2%, 9.20%)	− 29.5% (− 40.9%, − 15.1%)	− 39.1% (− 48.2%, − 28.8%)	46.1% (36.2%, 55.4%)
Age-standardised rate (percentage change, 2010–2019)	0.816% (− 7.52%, 9.95%)	9.01% (− 1.76%, 21.2%)	9.58% (− 3.02%, 23.6%)	2.05% (− 7.67%, 12.4%)	0.485% (− 5.66%, 5.91%)
*Deaths*					
Number (percentage change, 1990–2019)	133% (101%, 171%)	128% (94.1%, 164%)	84.6% (54.8%, 122%)	29.6% (10.5%, 51.4%)	201% (176%, 224%)
Number (percentage change, 2010–2019)	35.6% (24.7%, 47.2%)	45.9% (31.3%, 61.9%)	48.7% (31.4%, 66.4%)	28.8% (18.0%, 40.2%)	24.6% (17.1%, 30.9%)
Rate (percentage change, 1990–2019)	9.06% (− 5.73%, 26.7%)	46.1% (24.3%, 69.4%)	32.2% (10.9%, 59.3%)	4.21% (− 11.1%, 21.8%)	144% (124%, 162%)
Rate (percentage change, 2010–2019)	8.25% (− 0.421%, 17.6%)	29.6% (16.6%, 43.8%)	36.7% (20.8%, 53.0%)	22.1% (11.9%, 32.9%)	17.5% (10.5%, 23.4%)
Age-standardised rate (percentage change, 1990–2019)	3.78% (− 9.78%, 19.9%)	− 0.166% (− 14.9%, 15.6%)	− 22.0% (− 34.0%, − 6.54%)	− 32.5% (− 42.0%, − 21.9%)	60.6% (49.1%, 70.7%)
Age-standardised rate (percentage change, 2010–2019)	− 0.0300% (− 7.28%, 8.11%)	9.46% (− 1.78%, 21.8%)	10.4% (− 1.45%, 23.3%)	2.58% (− 6.11%, 11.6%)	1.47% (− 4.37%, 6.67%)

Data are presented as value (95% uncertainty interval)

### The temporal changes of LC incidence, death and DALYs attributed to NASH

**Fig. 1:**
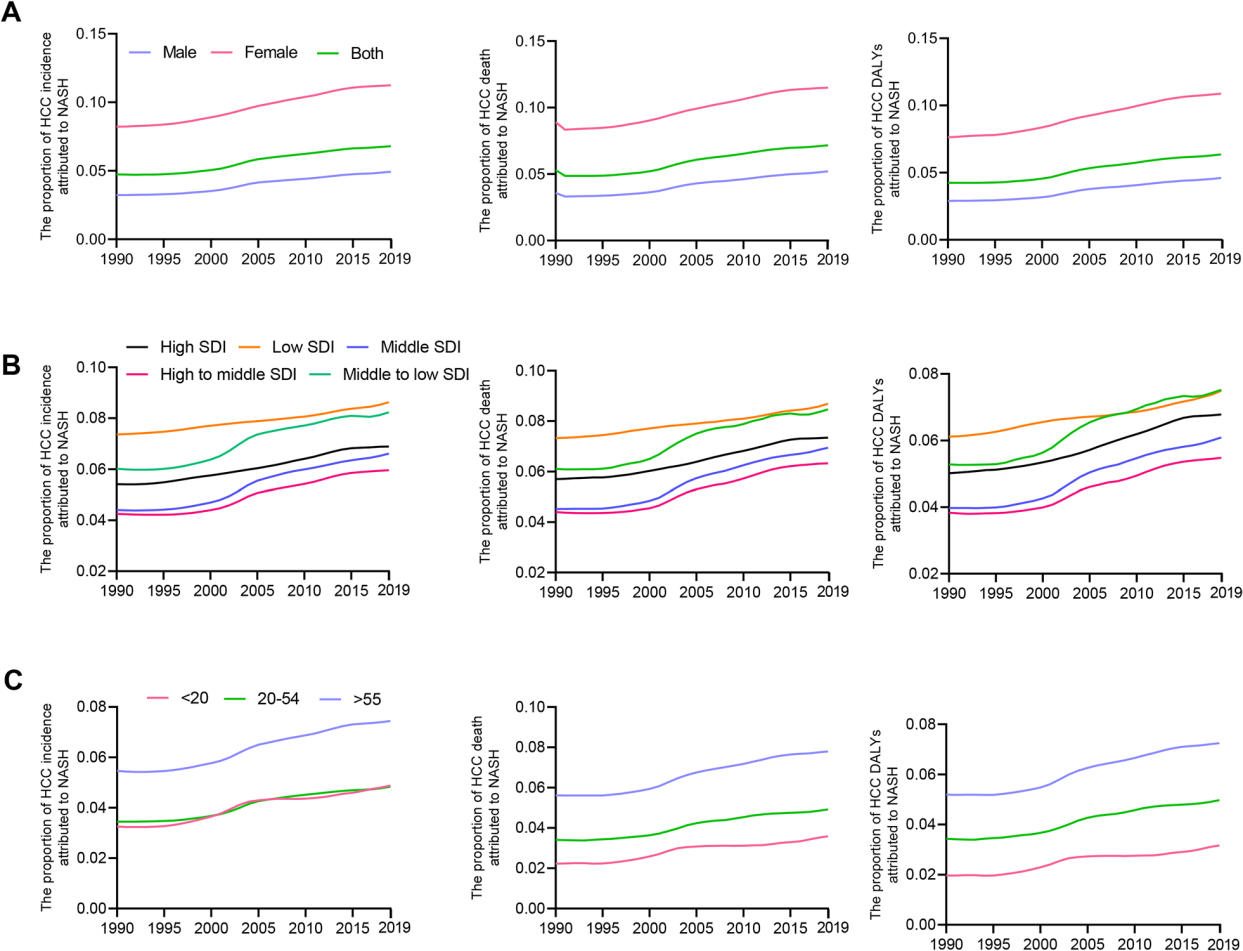
The temporal changes of LC incidence, death and DALYs attributed to NASH. **A** The temporal changes of LC incidence, death and DALYs attributed to NASH divided by gender. **B** The temporal changes of LC incidence, death and DALYs attributed to NASH divided by SDI locations. **C** The temporal changes of LC incidence, death and DALYs attributed to NASH divided by age

The proportion of LC incidences, deaths and DALYs attributed to NASH all increased from 1990 to 2019 (Fig. 1). The incidences, deaths and DALYs attributed to NASH were 4.74%, 5.30% and 4.25%, respectively in 1990 which were increased by 43.5%, 35.3% and 49.4%, respectively in 2019. And as shown in Fig. 1, the LC incidences, deaths and DALYs attributed to NASH were higher in female than male. Moreover, stratified by SDI, these five SDI locations all exhibit increased trend from 1990 to 2019 as for the NASH-related burden in total LC burden. When we divided the population into three age groups, we also found that all age groups showed obvious increase trend of NASH-related LC burden in total LC burden from 1990 to 2019. And among age groups, the age group of older than 55 years old had the highest proportion of LC burden attributed to NASH.

## Discussion

In this study, we analyzed and estimated the NASH-related LC burden globally over a 30-year period from 1990 to 2019 based on the GBD 2019 data. We found that the absolute numbers and rates of NASH-related LC incidence, mortality, and DALY significantly elevated from 1990 to 2019. Moreover, the incidences, deaths and DALYs of LC attributed to NASH also increased from 1990 to 2019. These results suggest that with the changes of lifestyle and pandemic of obesity and diabetes, the NASH-related LC burden is increasing significantly and needs to pay more attention to.

In 2019, the incidences, DALYs and deaths of NASH-related LC is just 36.3, 796 and 34.7 thousand globally. This number is not higher when compared to the burden of viral hepatitis related LC. And we found that the age-standardized incidence and mortality rates of NASH related LC did not change significantly from 1990 to 2019. However, the crude number of incidences, DALYs and deaths of NASH-related LC significantly increased by 105%, 66.2% and 95.1%, respectively in this period. The increase of crude number of NASH-related LC burden may be explained by the changes of demographic structure. NASH-related LC are more common in older people and in 2019, 703 million people were aged 65 years and older [6, 19]. Moreover, it is worthy to note that from 2010 to 2019, the age-standardized incidence rate of NASH related LC significantly increased by 8.16%, which suggests that the elevated trend of the NASH-related LC burden may be more obvious in recent years with significant higher prevalence of metabolic diseases [20].

Stratified by sex, we found that male people had higher incidences, prevalence, DALYs and deaths of NASH-related LC compared to female in 2019. Moreover, the crude number of incidences, DALYs and deaths of NASH-related LC increased more highly in male than female from 1990 to 2019. The higher NASH-related LC burden in male population may be due to higher LC rates in male as reported irrespective of etiology [21]. Because when we further investigated the proportion of LC burden attributed to NASH, the proportion of incidences, deaths and DALYs of LC attributed to NASH were much higher in female than male. NASH-related LC mostly occurs in elderly population. Estrogens have protective roles in the lipid metabolism and postmenopausal women are reported to develop metabolic disorder more easily [22, 23]. This may partially explain the higher proportion of LC burden attributed to NASH in female. Overall, our results demonstrate that male population had higher LC burden, but the female subjects had the higher proportion of LC burden attributed to NASH.

In the age-stratified analysis, the results showed that NASH-related LC was low in the people aged less than 20 years old. And with the age increased, the incidences, DALYs and deaths of NASH-related LC significantly elevated. These results are consistent with the findings that NASH-related LC tends to progress in older individuals [24]. The reasons of higher NASH-related LC in older population may be explained by following points. Firstly, the cancer initiation and progression to be detected need time and aging is a major risk factor for cancer development [25]. Secondly, the metabolic disorders burden such as diabetes and hyperlipidemias are heavier in older individuals than younger people [26]. The crude number of incident cases, DALYs and deaths increased significantly in individuals aged from 20 to 54 and older than 55 years old while there were no such changes in people aged less than 20 years old from 1990 to 2019. And we also can see that the incidence and mortality rate of NASH-related LC increased from 2010 to 2019 in individuals aged from 20 to 54 and older than 55 years old. These results suggest that NASH-related LC is now also significantly increased in the middle-aged individuals which requires attention to prevent the trend of rejuvenation of NASH-related LC.

Our results also revealed a large disparity of NASH-related LC burden in different SDI locations. In 2019, the crude number and the age-standardized rate of incidences, DALYs and deaths was highest in the middle SDI locations, while the rate of these indexes was highest in the high SDI locations. When investigated the temporal changes of the NASH-related LC disease burden, the crude number and rate of incidences, DALYs and deaths were increased significantly from 1990 to 2019 globally. These results were in line with the substantial increased number of metabolic disease populations globally in previous two decades [27]. Over the last 40 years, global lifestyles have changed in significant ways. Physical inactivity and unhealthy diet including consumption of hypercaloric meals and ultra-processed foods have become the key drivers of the metabolic disorders especially in developed countries [28]. As reported, the prevalence of NAFLD increased dramatically in the developed countries [29]. Consistent with the higher prevalence of metabolic disease and NAFLD, we found high SDI locations showed the largest increase of NASH-related LC burden from 1990 to 2019. As for age-standardized rate of incidence, DALYs and deaths, we also found a substantial increase in the high SDI locations from 1990 to 2019.

Our study has several limitations. Firstly, the accuracy of GBD estimates limited by the quality and quantity of data used in the modeling. For instance, misclassification of liver metastases to LC, underreporting of cancer and underestimation of LC caused by etiology. To challenge this limitation, the GBD group uses an integrative statistical model that combines all dimensions of related data to overcome the heterogeneity of bias and data source as much as possible [30]. Secondly,﻿ due to the lack of relevant data, the NASH-related LC did not stratify by histology, such as hepatocellular carcinoma. As the natural NAFLD progression course, we believe that most of NASH-related LC in our study are from hepatocellular carcinoma. Finally, the NASH-related LC prevalence is lower in this study compared to other studies. It may be due to how the GBD adjusted for alcohol use in the population [30, 31]. However, we still found a significant increase of NASH-related LC which is important information for policy maker to learn the trend of NASH-related LC.

## Data Availability

The datasets used and/or analysed during the current study are available from the corresponding author on reasonable request.

## Abbreviations

DALY: Disability-adjusted life years
GBD: Global Burden of Disease
HBV: Hepatitis B virus
HCC: Hepatocellular carcinoma
HCV: Hepatitis C virus
LC: Liver cancer
NAFLD: Nonalcoholic fatty liver disease
NASH: Nonalcoholic steatohepatitis
SDI: Socio-demographic index
YLD: Years lived with disability
YLL: Years of life lost
UI: Uncertainty interval
